# *Mycobacterium tuberculosis* Cluster with Developing Drug Resistance, New York, New York, USA, 2003–2009

**DOI:** 10.3201/eid1703.101002

**Published:** 2011-03

**Authors:** Bianca R. Perri, Douglas Proops, Patrick K. Moonan, Sonal S. Munsiff, Barry N. Kreiswirth, Natalia Kurepina, Christopher Goranson, Shama D. Ahuja

**Affiliations:** Author affiliations: New York City Department of Health and Mental Hygiene, New York, New York, USA (B.R. Perri, D. Proops, S.S. Munsiff, C. Goranson, S.D. Ahuja);; Centers for Disease Control and Prevention, Atlanta, Georgia, USA (B.R. Perri, P.K. Moonan, S.S. Munsiff);; Public Health Research Institute Tuberculosis Center, Newark, New Jersey, USA (B.N. Kreiswirth, N. Kurepina)

**Keywords:** Tuberculosis and other mycobacteria, Mycobacterium tuberculosis, bacteria, drug resistance, disease outbreak, contact tracing, molecular epidemiology, New York City, research

## Abstract

In 2004, identification of patients infected with the same *Mycobacterium tuberculosis* strain in New York, New York, USA, resulted in an outbreak investigation. The investigation involved data collection and analysis, establishing links between patients, and forming transmission hypotheses. Fifty-four geographically clustered cases were identified during 2003–2009. Initially, the *M. tuberculosis* strain was drug susceptible. However, in 2006, isoniazid resistance emerged, resulting in isoniazid-resistant *M. tuberculosis* among 17 (31%) patients. Compared with patients with drug-susceptible *M. tuberculosis*, a greater proportion of patients with isoniazid-resistant *M. tuberculosis* were US born and had a history of illegal drug use. No patients named one another as contacts. We used patient photographs to identify links between patients. Three links were associated with drug use among patients infected with isoniazid-resistant *M.* *tuberculosis*. The photographic method would have been more successful if used earlier in the investigation. Name-based contact investigation might not identify all contacts, particularly when illegal drug use is involved.

Name-based contact investigation is a core tuberculosis (TB) control method, yet its limitations are documented (*1**–**9*). Although name-based contact investigations can elucidate TB transmission chains, these investigations are typically limited to household and other close contacts (*10**,**11*). Molecular characterization of *Mycobacterium* *tuberculosis* (i.e., TB genotyping), when combined with contact investigation, can increase screening yield and identify transmission venues, particularly among populations at high risk (e.g., substance users, immigrants, and other hard-to-reach populations) (*2*–*5*,*12**,**13*).

Since 2001, the New York City (NYC) Bureau of Tuberculosis Control (BTBC), new York, New York, USA, has conducted universal genotyping and used results to detect and investigate clusters of TB with suspected recent transmission (*14*). One cluster, first identified and characterized in NYC in 2004, was the focus of an extensive epidemiologic investigation. We describe the investigation and discuss novel methods used during the investigation to understand TB transmission.

## Materials and Methods

Since January 1, 2001, all initial culture-positive *M. tuberculosis* isolates have been characterized by using spacer oligonucleotide type analysis (spoligotyping) at the New York State Department of Health Wadsworth Center (Albany, NY, USA) and IS*6110* restriction fragment length polymorphism (RFLP) typing at the Public Health Research Institute Tuberculosis Center (Newark, NJ, USA) (*14*). In accordance with Centers for Disease Control and Prevention (CDC) National Tuberculosis Genotyping Service, isolates were sent to the Michigan Bureau of Laboratories (Lansing, MI, USA) for 12-loci mycobacterial interspersed repetitive-unit variable-number tandem repeat (MIRU-VNTR) analysis (*15*).

### Case Definition

Cluster membership was defined as patients who had a diagnosis of TB in NYC during 2003–2009 and whose isolates had identical spoligotype and IS*6110* RFLP patterns. As the investigation continued, this definition was expanded and included patients whose isolates had identical spoligotype, 12-loci MIRU-VNTR results, and IS*6110* RFLP patterns with ± 1 band.

### Drug Susceptibility Testing

TB drug susceptibility testing (DST) was performed at the NYC Public Health Laboratory and the Wadsworth Center on initial *M. tuberculosis* isolates by using either BACTEC 460 or Mycobacterial Growth Indicator Tube 960 (Becton Dickinson, Sparks, MD, USA). A standard agar-proportion method with Middlebrook 7H10 media was used to confirm resistance (*16**–**18*). If DST indicated isoniazid resistance, DNA sequencing of the catalase–peroxidase G (*katG*) and enoyl reductase A (*inhA*) genes (*19*) was performed at the Public Health Research Institute Tuberculosis Center.

### Contact Investigation

Contact investigations were conducted per BTBC guidelines (*20*). For contacts of infectious index patients, staff assessed hours of TB exposure during infectious periods of patients, defined as the 12-week period before the patient began appropriate TB treatment (*20**,**21*). Infectious periods were extended to date of symptom onset if TB symptoms started >12 weeks before treatment began. Contacts having documented latent TB infection or TB symptoms were referred for medical evaluation and treatment.

### Cluster Investigation

An investigation of patients with the same *M. tuberculosis* strain was initiated to identify chains of transmission within the cluster and uncover epidemiologic links between TB patients. An epidemiologic link between 2 patients indicated that patients were linked by person, place, or time. Definite epidemiologic links between patients required 1 of the following criteria: named another patient as a contact, had a common contact, reported being in the same location during a patient’s infectious period, or recognized each other’s names or photographs. Probable epidemiologic links indicated that patients were in the same location during the same date range regardless of the infectious period of either patient or that 1 patient recognized another’s name or photograph. Possible epidemiologic links occurred when patients lived or visited an area within 0.8 km (0.5 miles) of another or had a similar social environment. If >1 link was established between 2 patients, the strongest link was counted.

Routine demographic and clinical data were obtained from the NYC TB registry and patient interviews. Additional data on homelessness and correctional history were obtained from NYC and New York State databases. Information regarding contacts and places of association (e.g., residences, worksites, and schools) of patients was analyzed to establish links between patients and to derive transmission hypotheses. To substantiate these hypotheses, we reinterviewed patients and their contacts by using a structured questionnaire. The questionnaire was updated with information obtained during patient interviews to ensure that hypotheses were reassessed throughout the investigation.

In October 2007, the NYC Department of Health and Mental Hygiene (DOHMH) Office of General Counsel approved use of names and photographs of patients and their contacts during interviews by BTBC. Cluster investigators administered informed consent forms. Consent forms indicated that names or photographs would be obtained and shown to persons being interviewed as part of the cluster investigation. If the patient denied voluntary permission but had an incarceration history, a public record booking photograph was used. To avoid disclosing confidential medical information, fictitious names and unrelated photographs were included in the compilation of names and photographs. Investigators did not confirm or deny a TB diagnosis of any person or how persons were related. During interviews, investigators asked if patients or contacts recognized any names or photographs. If recognition was indicated, the interviewer probed to understand how persons were linked.

### Statistical Analysis

We compared categorical data by using Pearson χ^2^ or Fisher exact tests, as appropriate. For continuous data, the Mann-Whitney test was used to compare medians. Statistical analyses were conducted by using SAS version 9.1 (SAS Institute, Inc., Cary, NC, USA).

Places of association were geocoded through the NYC Department of City Planning’s Geosupport Desktop Edition Software 9.6.9. Geocoded locations were imported into ArcGIS 9.2 (ESRI, Redlands, CA, USA) and mapped. Locations not geocoded by street address were geocoded by street intersection or other features. The ArcGIS point distance geoprocessing tool was used to calculate Euclidean distances between places of association of patients. Data were obtained as part of an outbreak investigation. Therefore, NYC DOHMH and CDC deemed this activity nonhuman subjects research.

## Results

**Figure 1:**
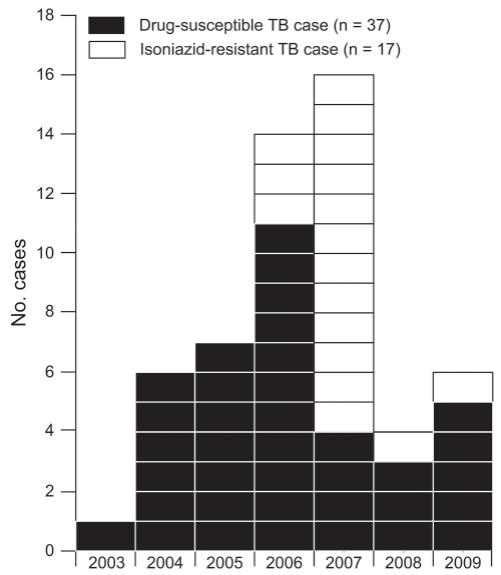
Cluster of 54 cases of tuberculosis (TB), by year of diagnosis, New York, New York, USA, 2003–2009. The 54 cases include 1 in a patient in the city of New York who was given a diagnosis of drug-susceptible *Mycobacterium tuberculosis* infection in 2007 that was counted by New York State.

**Figure 2:**
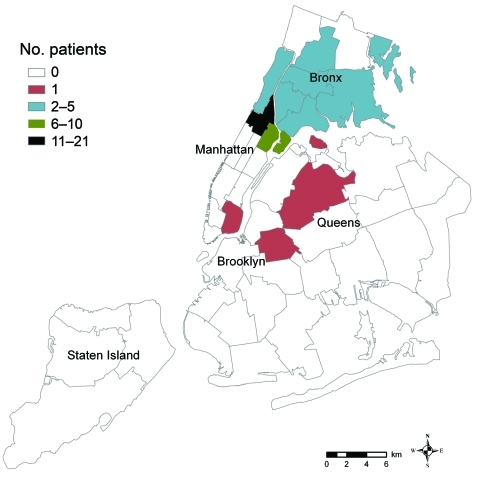
Residences of patients (n = 54) at time of tuberculosis diagnosis, by neighborhood, New York, New York, USA, 2003–2009. Forty-two neighborhoods were designated by the United Hospital Fund. Each neighborhood is defined by several adjoining ZIP codes (www.nyc.gov/html/doh/html/epi/mapgallery.shtml).

During 2003–2009, we identified 54 cases of TB as part of this cluster (Figure 1). Patient residence at TB diagnosis by NYC neighborhood is shown in Figure 2. Among 35 (65%) patients who lived in Upper Manhattan at diagnosis, median distance between the residence of any 2 patients was 1.4 km (range 0.01 km–6.6 km). Median distance between any 2 patients residing in the South Bronx (n = 10) at diagnosis was 2.9 km (range 0 km–5.8 km). Initially, the strain was susceptible to first-line anti-TB drugs. However, in 2006, isoniazid resistance emerged in a patient isolate at TB diagnosis. By 2009, 17 (31%) patients had isoniazid-resistant *M. tuberculosis* at diagnosis. All isoniazid-resistant isolates had the Ser315Thr mutation in the *katG* gene and no mutations in the *inhA* gene region sequenced.

### TB Genotyping

**Figure 3:**
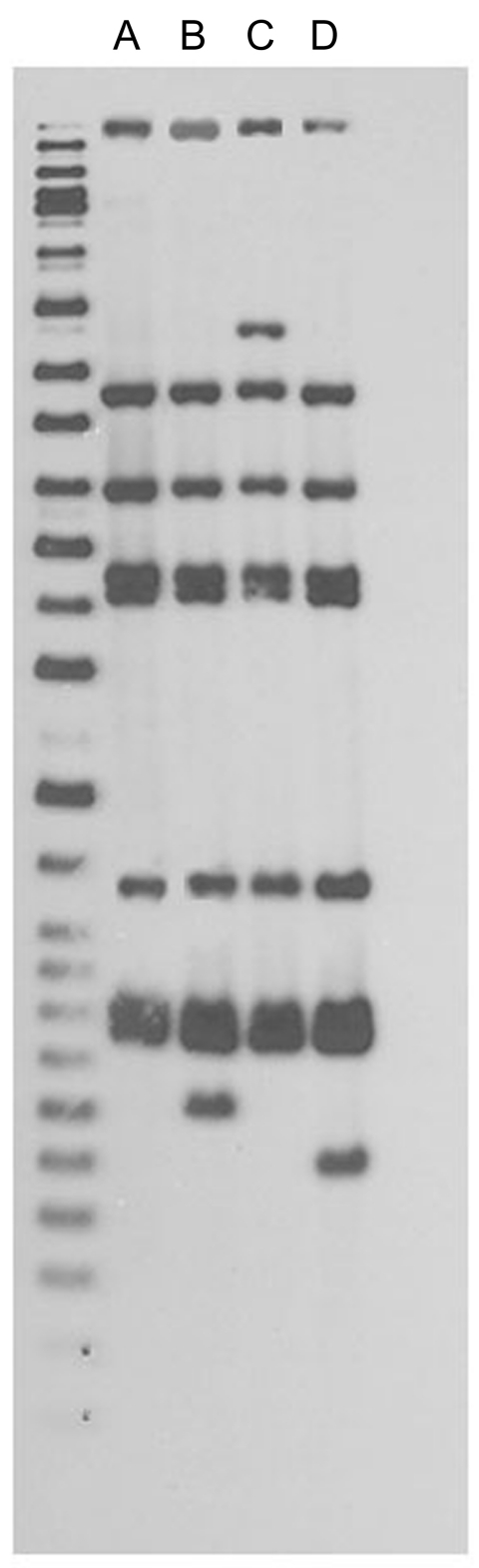
IS*6110* restriction fragment length polymorphism patterns for tuberculosis patients, New York, New York, USA, 2003–2009. Left lane, molecular mass ladder; lane A, n = 48; lane B, n = 1; lane C, n = 1; lane D, n = 4. Spoligotype results (octal code designation) were 777777774020771 for 54 patients. Twelve-loci mycobacterial interspersed repetitive-unit variable-number tandem repeat results were 225313153321 for 53 patients and 2253131–3321 for 1 patient; the dash indicates that there was no peak at this locus for this 1 patient, and the patient with this isolate met the original cluster case definition.

Forty-seven (87%) of the 54 patients had isolates with a matching spoligotype, IS*6110* RFLP pattern, and 12-loci MIRU-VNTR result (Figure 3). Forty-eight (89%) isolates met the original cluster case definition. Six (11%) were identified as cluster-associated patients on the basis of the expanded cluster case definition. As of December 31, 2008, within the CDC National Tuberculosis Genotyping Service database of 32,581 patient isolates, 6 with this cluster’s spoligotype and 12-loci MIRU-VNTR result were reported outside NYC (New York [n = 3], Delaware [n = 1], Georgia [n = 1], and Pennsylvania [n = 1]) (*22*). Among the 3 patients who resided in New York State, 1 was diagnosed in NYC and is therefore counted in the cluster (Figure 1); no link to NYC was identified for the other 2 patients.

### Patient Characteristics

Patient median age was 41 years (range 10–77 years); 74% were non-Hispanic black and 69% were male (Table). Among 37 patients with drug-susceptible *M.* *tuberculosis*, 73% were male and 38% were foreign born. The 17 patients with isoniazid-resistant *M. tuberculosis* were predominately US born (82%) and had a history of illegal drug use (59%) or incarceration (47%).

**Table:** Characteristics of 54 TB patients, by drug susceptibility test results, New York, New York, USA, 2003–2009*

Characteristic	All patients	Type of *Mycobacterium tuberculosis*		p value
		Drug-susceptible, n = 37	Isoniazid-resistant, n = 17	
Median age at TB diagnosis, y (range)	41 (10–77)	42 (12–77)	39 (10–52)	0.12
Male sex	37 (69)	27 (73)	10 (59)	0.30
Race/ethnicity				
Asian	1 (2)	1 (3)	0	1.00†
Hispanic	13 (24)	8 (22)	5 (29)	0.73†
Black, non-Hispanic	40 (74)	28 (76)	12 (71)	0.74†
Country of origin				
United States	36 (67)	22 (59)	14 (82)	0.10
Foreign	17 (31)	14 (38)	3 (18)	0.14
Unknown	1 (2)	1 (3)	0	1.00†
History of illegal drug use‡	22 (41)	12 (32)	10 (59)	0.07
History of homelessness	13 (24)	8 (22)	5 (29)	0.73†
History of incarceration	12 (22)	4 (11)	8 (47)	<0.01†
Pulmonary site of TB§	48 (89)	32 (87)	16 (94)	0.65†
Cavitary (among cases with pulmonary site of disease)	12 (25)	7 (22)	5 (31)	0.50†
Acid-fast bacilli smear positive for respiratory specimen	38 (70)	24 (65)	14 (82)	0.19
HIV status				
Positive	14 (26)	9 (24)	5 (29)	0.74†
Negative	37 (69)	26 (70)	11 (65)	0.68
Unknown	3 (6)	2 (5)	1 (6)	1.00†

*Values are no. (%) unless otherwise indicated. TB, tuberculosis. †By Fisher exact test. ‡Use of injection (e.g., heroin) or noninjection (e.g., marijuana or cocaine) drugs indicated on any patient record. §Includes patients with only pulmonary sites of disease and patients with pulmonary and extrapulmonary sites of disease.

**Figure 4:**
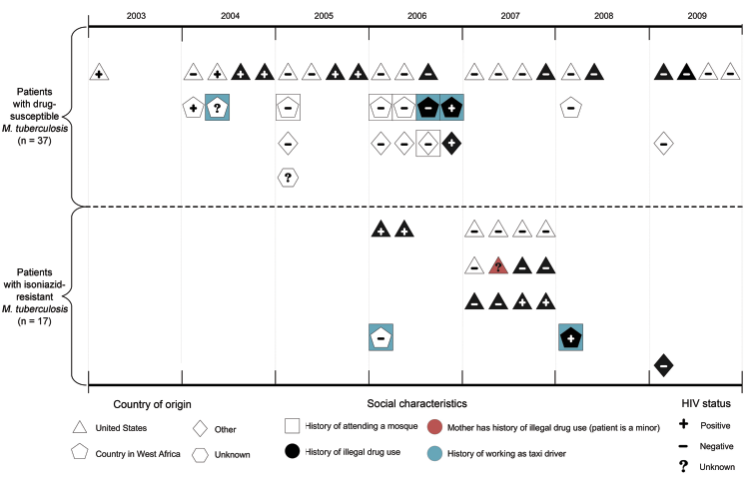
Common characteristics among 54 patients infected with *Mycobacterium tuberculosis*, by year of tuberculosis diagnosis and drug susceptibility testing results, New York, New York, USA, 2003–2009.

The shift of patient characteristics with time is shown in Figure 4. During 2003–2005, before isoniazid resistance emerged, 9 (64%) of 14 patients were US born and 4 (44%) of the US-born patients reported illegal drug use. Three patients, of whom 2 attended the same mosque, had a country of origin in West Africa; none reported drug use. In 2006, the number of patients with drug-susceptible *M. tuberculosis* peaked at 11, of whom 8 (73%) were foreign-born. All 4 patients from West Africa with drug-susceptible *M. tuberculosis* had a history of attending different mosques, and 2 had a history of illegal drug use. In 2007, when patients with isoniazid-resistant *M. tuberculosis* were more numerous than those with drug-susceptible *M.* *tuberculosis*, all 16 patients were US born; 8 (50%) were associated with illegal drug use. Of these 8 patients, 7 (88%) had isoniazid-resistant *M*. *tuberculosis*.

Among the 22 persons who disclosed a history of illegal drug use, 19 (86%) indicated noninjection drug use. The mother of a child with isoniazid-resistant *M*. *tuberculosis* also reported using illegal drugs. Drugs mentioned by patients connected to illegal drug use included smoking or snorting cocaine (n = 15), smoking marijuana (n = 6), and using heroin (n = 3).

### Contact Investigation

Among 48 patients eligible for contact investigation, 1,226 contacts were identified (median 9, range 0–153 contacts/patient). Twelve investigations of TB exposures in congregate settings were conducted. None of the clustered patients named one another as contacts. Contact investigation identified 1 clinically diagnosed TB case linked to a cluster-associated patient with drug-susceptible *M. tuberculosis*.

### Cluster Investigation

All 2-patient combinations (n = 1,431) were analyzed for epidemiologic links. Routine cluster investigation identified 3 definite epidemiologic links; only 1 of these links involved a patient with isoniazid-resistant *M. tuberculosis*. One definite epidemiologic link was based on a common contact between a patient with drug-susceptible *M. tuberculosis* and a patient with isoniazid-resistant *M. tuberculosis*. The other 2 links were based on patients living in the same apartment building during the infectious period of 1 of the patients. Cluster investigation methods identified 3 probable epidemiologic links; all involved patients attending the same mosque during an overlapping date range. All 54 patients had a possible epidemiologic link to at least 1 other cluster patient; 98% of patients had multiple possible epidemiologic links. Of the possible epidemiologic links identified, 81% were geographic and 29% involved illegal drug use. Other possible epidemiologic links were identified on the basis of shared patient characteristics such as having a country of origin in West Africa, being infected with HIV, and history of mosque attendance, taxi driver occupation, incarceration, or homelessness.

During 2007–2009, patients were asked for permission to use names and photographs. Ten (59%) of the 17 patients with isoniazid-resistant *M. tuberculosis*, 1 (17%) of 6 patients with drug-susceptible *M. tuberculosis*, and 7 (64%) of 11 contacts granted permission. Public-record booking photographs were used for 2 patients. Four additional probable epidemiologic links were established through name and photograph use; all were associated with illegal drug use. Patients did not indicate familiarity with fictitious names and unrelated photographs that were presented.

## Discussion

Despite using substantial resources within BTBC and beyond, we did not clearly identify chains of transmission in this outbreak. Only 3 definite epidemiologic links were identified between patients, and only 1 was associated with the rapidly emerging or spreading isoniazid-resistant strain. The strongest link of this cluster is geographic; patients primarily spent time in the same neighborhoods. Although matching genotype does not always signify recent transmission, geospatial concentration and epidemiologic data indicate ongoing and recent transmission of this rare genotype in NYC. Contact investigation results showed evidence of possible transmission. However, no confirmed secondary TB cases were identified among >1,200 identified contacts, further demonstrating limitations of name-based contact investigation.

This outbreak was only identified through genotyping. PCR-based methods (spoligotyping and 12-loci MIRU-VNTR analysis) better defined this TB cluster. Supplementing contact investigation with laboratory tools to examine strain relatedness (e.g., real-time genotyping and DST) can help TB control program staff identify and investigate outbreaks. Although all patient specimens had a matching genotype, DST results showed 2 phenotypes, and therefore >2 distinct transmission chains within the cluster. Identifying separate transmission chains enabled cluster investigators to develop and test hypotheses specific to each chain of transmission. Common characteristics within each transmission chain implied discrete social networks, but these networks could not be confirmed by using routine cluster investigation methods.

Emergence of isoniazid resistance in this cluster cannot be clearly explained. None of the patients with drug-susceptible *M. tuberculosis* showed failure of treatment. Presumably, 1 person, identified by investigators as a shared contact between a patient with drug-susceptible *M. tuberculosis* and a patient with isoniazid-resistant *M.* *tuberculosis*, had a history of taking medications for TB and showed development of isoniazid-resistant *M. tuberculosis* that had not been reported to BTBC. This person died; therefore, cluster investigators were unable to confirm this hypothesis despite medical record review and pharmacy surveillance.

This investigation was limited by patients’ unwillingness to report their contacts, possibly because of fear of disclosing immigration status (not asked by BTBC staff), illegal drug use, or involvement in other illicit activities. Other possible explanations include forgetting or not knowing their contacts by name (*2**,**23*). Certain patients used aliases (not tracked in the NYC TB registry) and claimed to only know their contacts by first names or aliases. Pervasiveness of aliases within patient social networks stymied contact investigation efforts and made establishing epidemiologic links between patients difficult.

High prevalence of illegal drug use within the cluster led investigators to explore how specific drug-use practices contribute to TB transmission. Studies reported that such specific drug-use practices as shotgunning (inhaling smoke from rock cocaine or marijuana and blowing the smoke directly into the mouth of another) and hotboxing (smoking drugs in a small, enclosed space to maximize narcotic effect through first-hand and second-hand smoke) were associated with TB transmission (*24**,**25*). Although these practices were not specifically mentioned by patients or their contacts, specific questions were not asked until later in the investigation. After consulting with substance-use experts, BTBC revised their cluster-investigation questionnaire and provided investigators with additional training on patient-interview procedures and drug-use subculture. Understanding drug-use behavior helps TB control personnel elicit sensitive transmission information. BTBC also modified how substance-use information is collected and recorded in the TB registry.

Transmission through casual contact and increased virulence are possible explanations for extensive transmission of this strain and lack of recognition among patients. Although TB transmission from casual contact is considered rare, it has been documented (*26**–**30*). If this strain, like other outbreak strains (*29*), was highly virulent, extensive transmission among patients who did not recognize each other would have been possible. Moreover, geographic proximity of patients to one another might have increased opportunities for TB exposure and supported transmission through casual contact. In addition, positive results for acid-fast bacilli in smears of respiratory specimens among cluster-associated patients were substantial (70% overall, 93% among cocaine users) and considerably greater than recent past NYC TB patients (range 42%–46% during 2003–2008) (NYC DOHMH, unpub. data), thus increasing likelihood of transmission. Investigation findings were consistent with those of a London study that reported that pulmonary TB patients who used cocaine were more likely to be sputum smear positive at diagnosis (*31*), perhaps related to delays in seeking medical care.

Photograph and name use yielded the strongest epidemiologic links between patients with isoniazid-resistant *M. tuberculosis*. It was the only method that confirmed patient recognition within the cluster. All epidemiologic links established through photograph recognition were related to illegal drug activity. Other outbreak investigations have highlighted unwillingness of patients to share social contacts when these contacts are connected to illegal activities (*4**,**5**,**13*).

Insights gained from using name and photograph data in an ongoing investigation will benefit TB control programs. This method would have been more successful if used earlier in the investigation. TB control personnel contemplating adopting this strategy should obtain legal guidance before an outbreak occurs because privacy laws vary from one locality to another.

This outbreak investigation highlights an array of challenges for US-based TB control programs. Understanding and preventing TB transmission among hard-to-reach populations requires considerable resources. Conventional contact investigation can be inadequate for identifying and curtailing TB transmission among difficult-to-reach- populations. New methods, including using name and photograph data, are needed for TB elimination.
